# Functional Recovery after Scutellarin Treatment in Transient Cerebral Ischemic Rats: A Pilot Study with ^**18**^F-Fluorodeoxyglucose MicroPET

**DOI:** 10.1155/2013/507091

**Published:** 2013-05-02

**Authors:** Jin-hui Li, Jing Lu, Hong Zhang

**Affiliations:** ^1^Department of Nuclear Medicine, Second Affiliated Hospital of Zhejiang University School of Medicine, Hangzhou, Zhejiang 310009, China; ^2^Department of Traditional Chinese Medicine & Rehabilitation, Second Affiliated Hospital of Zhejiang University School of Medicine, Hangzhou, Zhejiang 310009, China; ^3^Zhejiang University Medical PET Center, Hangzhou, Zhejiang 310009, China; ^4^Institute of Nuclear Medicine and Molecular Imaging of Zhejiang University, Hangzhou, Zhejiang 310009, China; ^5^Key Laboratory of Medical Molecular Imaging of Zhejiang Province, Hangzhou, Zhejiang 310009, China; ^6^Department of Neurobiology, Zhejiang University School of Medicine, Hangzhou, Zhejiang 310058, China

## Abstract

*Objective*. To investigate neuroprotective effects of scutellarin (Scu) in a rat model of cerebral ischemia with use of ^18^F-fluorodeoxyglucose (^18^F-FDG) micro positron emission tomography (microPET). *Method*. Middle cerebral artery occlusion was used to establish cerebral ischemia. Rats were divided into 5 groups: sham operation, cerebral ischemia-reperfusion untreated (CIRU) group, Scu-25 group (Scu 25 mg/kg/d), Scu-50 group (Scu 50 mg/kg/d), and nimodipine (10 mg/Kg/d). The treatment groups were given for 2 weeks. The therapeutic effects in terms of cerebral infarct volume, neurological deficit scores, and cerebral glucose metabolism were evaluated. Levels of vascular density factor (vWF), glial marker (GFAP), and mature neuronal marker (NeuN) were assessed by immunohistochemistry. *Results*. The neurological deficit scores were significantly decreased in the Scu-50 group compared to the CIRU group (*P* < 0.001). ^18^F-FDG accumulation in the ipsilateral cerebral infarction increased steadily over time in Scu-50 group compared with CIRU group (*P* < 0.01) and Scu-25 group (*P* < 0.01). Immunohistochemical analysis demonstrated Scu-50 enhanced neuronal maturation. *Conclusion*. ^18^F-FDG microPET imaging demonstrated metabolic recovery after Scu-50 treatment in the rat model of cerebral ischemia. The neuroprotective effects of Scu on cerebral ischemic injury might be associated with increased regional glucose activity and neuronal maturation.

## 1. Introduction

Positron emission tomography (PET) is widely used for the clinical evaluation of neurological disease. The ^18^F-fluorodeoxy-D-glucose (^18^F-FDG) method has been used for quantitative measurement of glucose utilization. In our previous study, we have measured the ischemic damage in the brain by means of ^18^F-FDG and demonstrated that a PET imaging scan might serve as a noninvasive therapeutic followup for individual animals [1]. We previously used alternative medicine therapies, including herbs and formulas, to examine cerebral ischemic reperfusion [2]. However, the increasing use of traditional therapies demands more scientific evidence for the principles behind them and for their effectiveness. Scutellarin (Scu), the main bioactive component of *Erigeron breviscapus*, has been used in the treatment of cerebral and heart vascular diseases [3, 4]. Moreover, it also inhibits the replication of several strains of HIV-1 [5] and diminishes hyperglycemia [6]. A preliminary study revealed that neuroprotection by Scu is mediated by blocking the sodium current of hippocampal neurons [7], by inhibiting the formation of hydrogen peroxide and the neuroinflammatory reaction of microglia [8] and apoptosis-inducing pathways [9], and by increasing constitutive nitric oxide synthase (cNOS) [10]. Many brain injuries, such as cerebral ischemia [11–13], hypoxia [14], and hemorrhage [15], are associated with the proliferation of endogenous neural progenitor cells. The exact function of these cells and their fate following cerebral ischemic reperfusion is still not clear [16]. Therefore, we aimed to monitor neural progenitor cells for a longer period after ischemic stroke in adult rat brain [11, 17, 18]. However, the recovery of neurological function mainly depends on the neurovascular unit (NVU), that is, on interactions between astrocytes, brain microvessels, and their environment [19], all of which may exert either protective or harmful actions.

NVU as a paradigm [20, 21] is broadly applicable to cerebral ischemia. The basement membrane that underlies the endothelial cells is a key structure for maintaining the integrity of the neurovascular unit, and a free radical scavenger can be a viable agent for inhibiting tissue plasminogen activator [22]. Glial scarring is commonly thought to have an adverse effect after stroke. However, a new study now suggests that reactive astrocytes in the peri-infarct cortex may additionally contribute to neurovascular remodeling [23]. Stroke-generated new neurons and neuroblasts that were probably already present before the insult migrated into the severely damaged area of the cortex, where they express markers of developing, mature, and striatal-like spiny neurons [24]. We tested the hypothesis that cerebral ischemic damage prolongs the impairment of the metabolic coupling of neurovascular units and neural cells. Impaired neurovascular coupling was explained by reduced vascular reactivity and suppressed function of cortical inhibitory interneurons. Intriguingly, some flavonoids such as galangin, chrysin, and pinocembrin have the capacity to shield the neurovascular unit in rats [25] and ensure neuroprotection against cerebral ischemia/reperfusion (I/R) injury [20]. Moreover, we speculate that neurovascular plasticity is characterized by increasing regional glucose activity and neuron numbers that provide protection against stroke weeks longer than previously established. 

To better understand the mechanisms involved and to be able to make proposals for the prevention of cerebral I/R injury, as well as give a prognosis, further study is required. Scu might have bidirectional regulation effects, as well as being able to dispose of paradoxical diseases, like hemorrhagic cerebral infarction simultaneously [26, 27]. Therefore, the present study mainly explored the effects of Scu on neuronal regeneration, astrocyte activity, and microvascular and regional glucose activity in transient middle-cerebral artery-occlusion- (MCAO-) induced focal cerebral ischemia in rats. 

## 2. Materials and Methods 

### 2.1. Animals, Experimental Groups, and Outline of the Experiment

Adult male SD rats weigh 250–280 g with the light-dark cycle each 12 hours, filtrate water, and total nutrient feed. Then, all rats were divided into five experimental groups (12 rats for each group): sham group, a cerebral ischemia-reperfusion untreated (CIRU) group, Scu-25 group (Scu 25 mg/Kg/d), Scu-50 group (Scu 50 mg/Kg/d), and nimodipine group (10 mg/Kg/d). All the groups were fed by oral gavage feeding for a period of 2 weeks. Behavioral tests and PET imaging were performed at 1, 7, 14, 21, and 28 days after MCAO. All rats were euthanized at the 28 days after MCAO for immunohistochemical detection.

### 2.2. Animal Model of Middle Cerebral Artery Occlusion (MCAO)

All rats were anesthetized by injections of 1.5% pentobarbital sodium (50 mg/kg, intraperitoneal). Focal brain ischemia was induced by the intraluminal suture. A midline skin incision in the neck was followed by subsequent exploration of the right common carotid artery (CCA), the external carotid artery (ECA), and the internal carotid artery (ICA). A 4-0 monofilament nylon suture with a rounded tip was introduced into the left internal carotid by arteriotomy and advanced 18–20 mm past the carotid bifurcation. After 1 h of MCAO, reperfusion was achieved by withdrawing the endovascular suture to the stump of the ECA, and the skin was then sutured [28]. Rectal temperature was monitored continuously and maintained at 36.5–37.5°C for the duration of the surgery. The rats were housed in environmentally enriched conditions under a 12 h light/12 h dark cycle throughout the experiments and were allowed free access to food and water.

### 2.3. Neurological Functional Tests

A modified neurological severity score (mNSS) test was performed [29], post-MCAO at 24 h and at 1, 2, 3, and 4 weeks after reperfusion. The mNSS was graded on a scale of 0 to 18 (normal score, 0; maximal deficit score, 18). In the severity scores of injury, 1 score point is awarded for the inability to perform the test or for the lack of a tested reflex; thus, the higher the score, the more severe the injury.

### 2.4. Triphenyltetrazolium Chloride Assessment

Total 15 rats (each group, *n* = 5) were anesthetized and sacrificed by rapid decapitation. The rat brain was collected at 7 d after reperfusion. The brain was kept at −20°C, then sliced into 2 mm thick coronal sections and stained with 1% 2,3,5-triphenyltetrazolium chloride (Sigma Chemicals) solution for 15 min at 37°C, and fixed in 4% paraformaldehyde at 4°C overnight. After fixation, the brain slices were scanned using a flat-bed scanner. Infarct volume was quantified with professional image analysis software (Analysis Life Science Professional). Infarct volumes were calculated by adding up the infarct areas in six brain slices by thickness (2 mm) slightly.

### 2.5. MicroPET Imaging and Data Analysis

At 24 hours after reperfusion and at 1, 2, 3, and 4 weeks after MCAO, rats were anesthetized with isofluorane (2%) and injected with approximately 18.5 MBq of ^18^F-FDG via the tail vein. At 30 min after ^18^F-FDG injection, rats were anesthetized with isofluorane (2%), and a 10 min static acquisition was performed with a midhead machine, and images were reconstructed using a maximum a posteriori algorithm. Uptake was calculated as the percentage of injected dose per gram (%ID/g) of tissue using the ASIPro 6.0.5.0 software (CTI Concorde Microsystems, LLC profile in Knoxville, TN, USA) at the Medical PET Center of Zhejiang University. Then, rats were anesthetized with isoflurane and prone positioned on the microPET R4 rodent model scanning gantry (Siemens Preclinical Solutions, Knoxville, TN, USA). The scanner has a computer-controlled bed and 10.8 cm transaxial and 8 cm axial fields of view. The voxel size was 0.845 mm on a side, and the full width at half maximum was 1.66, 1.65, and 1.84 mm for tangential, radial, and axial orientations, respectively. In each scan, 3 different regions of interest (ROI), 0.5 mm in diameter manually drawn by creating a volume of interest in the central of the ischemic area and using the mean dose per gram of tissue (%ID/g), were averaged. The lesion-to-normal homologous cerebellum ratio (L/N) was used for semiquantitative analysis. L/N ratio was calculated using the following formula: L/N ratio = mean counts per pixel of lesion ROIs/mean counts per pixel of normal homologous cerebellum ROIs.

### 2.6. Immunohistochemical Assessment

Animals were overdosed with 10% chloral hydrate, after which their thoracic cavities were opened and perfused intracardially with normal saline followed by 4% paraformaldehyde in 0.1 M phosphate buffer (pH 7.4). The brain was removed and fixed by immersion in fresh fixative overnight and stored in 30% (v/v) phosphate-buffered sucrose overnight. Differentiation of the nature of cells was determined by colocalizing markers for different cell types (vWF: rabbit polyclonal antibody, DAKO A008229,1:200; GFAP: rabbit polyclonal antibody, Abcam ab7260,1:300; NeuN: mouse monoclonal antibody, clone: A60, Millipore MAB377, 1:200). Sections were blocked and incubated overnight at 4°C with primary antibodies. After having been washed in PBS, sections were incubated with conjugated secondary antibodies (antimouse or antirabbit Alexa 568) for 1 h at 37°C following 2-3 min rinsing in PBS with diaminobenzidine chromagen (0.5 mg/mL), rinsed twice in distilled water, 50 mL hematoxylin stained for 2–5 min, differentiated with 1% hydrochloric acid alcohol, and dehydrated with dimethylbenzene transparent, blue-based cover slipped with glycerin (Shanghai Sengene biotech Co., Ltd.), after which the sections were examined under a light microscope and IODs of vWF, GFAP, and NeuN quantitative analysis by using Image-Pro Plus 5.0 software (Media Cybernetics).

### 2.7. Statistical Analysis

All results were expressed as mean ± SEM accompanied by the number of observations. Continuous variables were compared by the unpaired Students *t*-test, and multiple groups were compared by ANOVA using the* Bonferroni *correction. Significant differences were accepted when *P* < 0.05.

## 3. Results

### 3.1. Effects on Neurological Deficit

**Figure 1:**
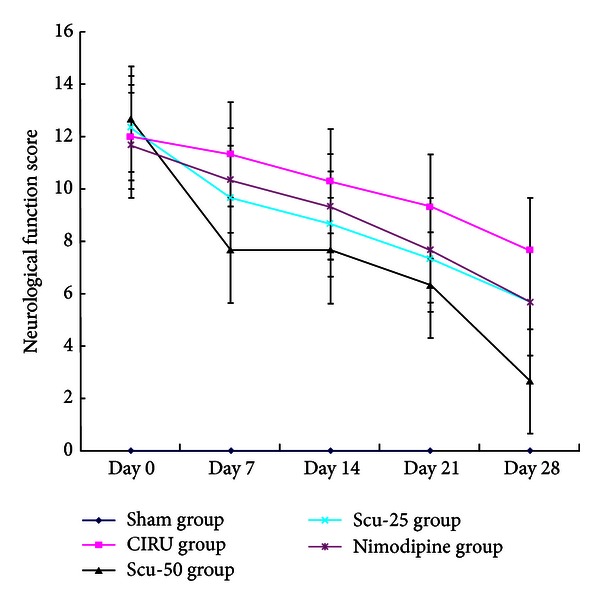
Neurological functional tests to describe rat neurological scores of middle cerebral artery occlusion-induced focal cerebral ischemia. Sham group (sham), cerebral ischemia-reperfusion untreated group (CIRU), scutellarin 50 mg/Kg group (Scu-50), scutellarin 25 mg/Kg group (Scu-25), and nimodipine 10 mg/Kg group (Nimodipine) in days 0, 7, 14, 21, and 28, respectively.

After MCAO, the neurological deficit score of the CIRU group was significantly higher than that of the sham group (*P* < 0.01) on days 0, 7, 14, 21, and 28, whereas those of the Scu-50 group (50 mg/kg/d) and Scu-25 group (25 mg/kg/d) were lower than those of the occluded group on day 7 and day 14 (*P* < 0.001, *P* < 0.05 separately). The scores of the Scu-50 group (*P* < 0.001), Scu-25 group (*P* < 0.01), and nimodipine group (*P* < 0.05) were significantly lower than that of the model group on day 21. And the scores of the Scu-50 group (*P* < 0.001), Scu-25 group (*P* < 0.05), and nimodipine group (*P* < 0.05) were lower than that of the occluded group on day 28. Furthermore, the scores of the Scu-50 group were lower than those of the Scu-25 group and nimodipine group (*P* < 0.01) (Table 1 and Figure 1). 

### 3.2. Triphenyltetrazolium Chloride Evaluation

**Figure 2:**
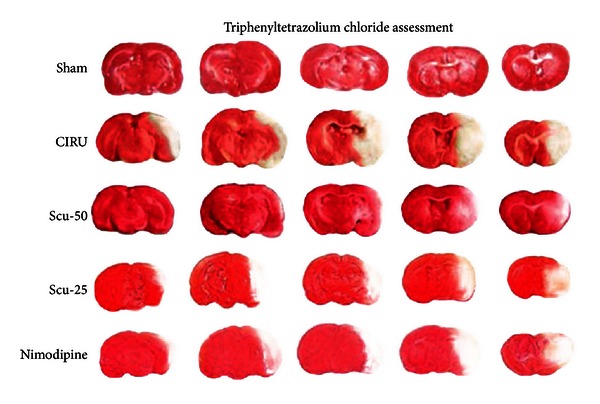
2,3,5-Triphenyltetrazolium chloride (TTC) staining to delineate rat brain infarcts of middle cerebral artery occlusion-induced focal cerebral ischemia. Sham group (sham), cerebral ischemia-reperfusion untreated group (CIRU), scutellarin 50 mg/Kg group (Scu-50), scutellarin 25 mg/Kg group (Scu-25), and nimodipine 10 mg/Kg group (nimodipine) at day 14 after reperfusion.

The infarct volume in the occluded group was significantly increased compared to the sham group (*P* < 0.001). The Scu-50 group showed the smallest infarct volume (56.0 ± 2.00 mm^3^, *P* < 0.001), followed by the Scu-25 group (74.3 ± 3.51 mm^3^, *P* < 0.001) and the nimodipine group (98.3 ± 2.06 mm^3^, *P* < 0.001), when compared with the CIRU group (244.3 ± 7.76 mm^3^) (Figure 2 and Table 2).

### 3.3. Effects on Brain Glucose Metabolism

**Figure 3:**
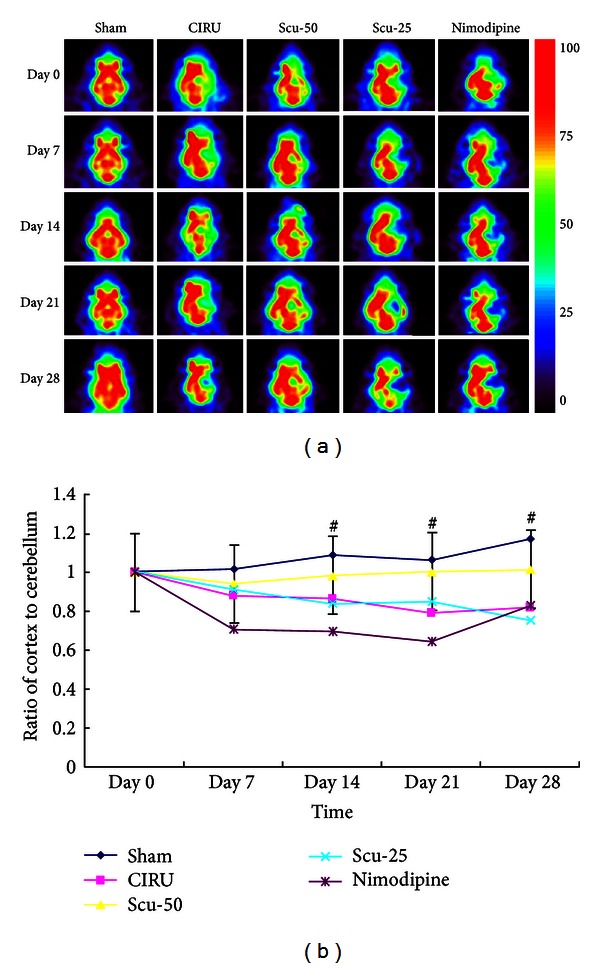
(a) ^18^FDG-PET images of activity of cerebral ischemic regions of the glucose metabolism. Sham group (sham), cerebral ischemia-reperfusion untreated group (CIRU), scutellarin 50 mg/Kg group (Scu-50), scutellarin 25 mg/Kg group (Scu-25), and nimodipine 10 mg/Kg group (nimodipine) in days 0, 7, 14, 21, and 28 respectively. (b) Ratios of cortex to cerebellum in different regions in the brain of the glucose metabolism sham group (sham), cerebral ischemia-reperfusion untreated group (CIRU), scutellarin 50 mg/Kg group (Scu-50), scutellarin 25 mg/Kg group (Scu-25), and nimodipine 10 mg/Kg group (nimodipine) in days 0, 7, 14, 21, and 28, respectively.

Glucose metabolism in the brain was significantly reduced in the damaged region of the CIRU group compared with the sham group (*P* < 0.01). Glucose metabolism of Scu-50 group was increased significantly (*P* < 0.01) on day 14 compared with the CIRU group, whereas there was no significance of the Scu-25 group and the nimodipine group in the right cerebral ischemic regions compared with the CIRU group on days 7, 14, 21, and 28. Intriguingly, ^18^F-FDG uptake of focal ischemic lesion in Scu-50 was also higher than Scu-25 group (Table 3). Furthermore, the Scu-50 group yielded the best glucose metabolism increasing compared with CIRU group, Scu-25 group, and nimodipine treatment groups (Figure 3). 

### 3.4. Expression of Vascular Density Factor (vWF), GFAP, and NeuN Immunoreactivity

**Figure 4:**
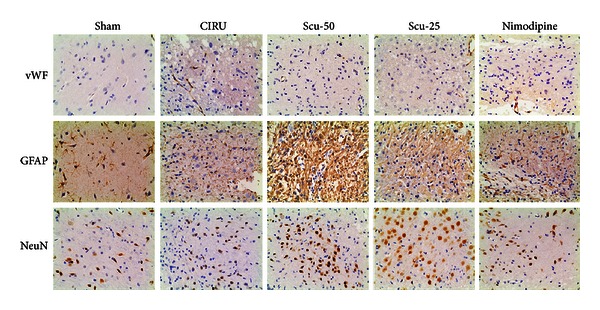
Vascular density factor (vWF), mature glial marker (GFAP), and neurogenesis markers (NeuN) immunostained tissue (magnification 200x) of middle cerebral artery occlusion-induced focal cerebral ischemia in rats. Sham group (sham), model group (CIRU), scutellarin 50 mg/Kg group (Scu-50), scutellarin 25 mg/Kg group (Scu-25), and nimodipine group (nimodipine) at day 28 after reperfusion.

Immunohistochemical studies were performed to determine whether Scu treatment can induce neurogenesis and angiogenesis. NeuN was used as a mature neuronal marker, GFAP as the mature astrocytes marker, and vWF as the endothelial cell marker. We counted the number of cells immunostaining positively the NeuN or vWF and calculated the mean values and SEMs. All the data of immunochemistry analysis were summarized in Figure 4 and Table 4. For the vWF expression, immunoreactivity was higher in the CIRU group and treated groups than in the sham group (*P* < 0.05). GFAP-positive cell number was significantly increased in the 50 mg/Kg Scu and 25 mg/Kg Scu groups compared with the sham group (*P* < 0.05). However, all the Scu groups showed no significance compared with the CIRU group. Expression of NeuN intensity in the Scu-50 group significantly increased compared with the CIRU group (*P* < 0.001). NeuN intensity in the Scu-50 group also increased significantly compared with the nimodipine group (*P* < 0.05). Interestingly, the Scu-50 group showed a significant higher expression of NeuN immunoreactivity compared with the Scu-25 group (*P* < 0.05).

## 4. Discussion

Following cerebral ischemia, the extracellular concentration of excitatory amino acids increases, resulting in the excitatory cell death in ischemic neuronal damage [30]. Although sequential metabolic changes in permanent cerebral ischemia have been reported [31], the effect of reperfusion in local cerebral ischemia on glucose metabolism is less clear. In order to study the time course of the changes in glucose metabolism following middle cerebral artery occlusion-reperfusion model and the effects of Scu on glucose metabolism, the ^14^C-deoxyglucose method was used [32]. Hypermetabolism occurred at 30 minutes after the middle cerebral artery (MCA) occlusion and reached a peak at 60 min after ischemia in both ischemic core and penumbra regions [33]. 

*Breviscapine*, one plant flavonoids found from *Erigeron breviscapus*, could dilate brain blood vessels, increased cerebral blood flow and cardiac coronary flow, reduce blood viscosity, and improve microcirculation [34]. Traditionally it has been used for thousand years. We estimated that the main ingredient of *Erigeron breviscapus*, Scu, might inhibite both increased glucose metabolism during ischemia and decreased glucose metabolism during reperfusion. These findings support the hypothesis that excitation-induced hypermetabolism plays a major role in the ischemic insult following focal cerebral vascular occlusion [35, 36]. 

Scu, one of the ingredients of *breviscapine*, was used for neuronal damage. This compound was tested for increasing [Ca^2+^] ion and activating protein kinase C*γ* (PKC*γ*) following cerebral ischemia and reperfusion [37] and was also found to play a vital role in protecting PC12 cells from cobalt chloride-induced apoptosis by scavenging reactive oxygen species, inhibiting p38 phosphorylation, upregulating Bcl-XL expression, and decreasing caspase-3 activity [38]. Moreover, Scu attenuated H_2_O_2_-induced cytotoxicity, lipid peroxidation, and loss of DNA [39] and inhibited hydrogen peroxide increased activity of cNOS and oxidative damage induced by superoxide in synaptosomes [40]. Also, it showed an inhibition of polyadenosine diphosphate ribose polymerase-dependent mitochondrial dysfunction and subsequent translocation of apoptosis-inducing factor *in vitro* [41]. In this study, compared with the control group, Scu (50 mg/kg) reduced neurons apoptosis in cerebral infarction rats, and a dose-dependent Scu influence degree of the effects on anticerebral ischemia was observed *in vivo* [42]. 

Our data confirmed that the dose-dependent increased brain glucose metabolism is as observed earlier in brain PET studies, since the cerebellum does not show alterations under the circumstances [43, 44] and the ratio of lesion to cerebellum region index is more commonly used in PET studies [36, 45]. During the delayed phase, brain angiogenesis may provide the critical neurovascular substrates for neuronal renew. As we know, ^18^F-FDG uptake and regional cerebral blood flow represent neuron activity both *in vitro* and *in vivo *[46]. Although the ratio of ischemic cortex to nonischemic cortex has no consistency to the actual effect, it is not influenced by the weight of the rat or the amount of radionuclide flow at the injection points. So we chose the ratio of ischemic cortex to nonischemic cerebellum which is more stable than the ratio of the lesion/contralateral cortex region of brain glucose metabolism. One result showed that glucose metabolism utilization in cerebellum decreased at all times in special neurons of focal ischemia rat, which was not totally consistent with our results [47]. 

Scu, also called chelerythrine chloride, was considered the PKC inhibitors, which have potential as anti-ischemia agents *in vitro* (IC50 = 0.66 *µ*M) and *in vivo* [48, 49]. And as we know PKC*γ* has the most abundant distribution in the cerebellum, hippocampus, and cerebral cortex [50]. We speculated that Scu primarily and specially blocks ischemic tissues in rat brain by activating PKC*γ* and exerting protective effects against cerebral ischemia-reperfusion injury *in vivo*. 

Because the expression of vWF activity was higher in the CIRU group and treatment groups than in the sham group, we presume to focal ischemia, the cerebral microvasculature multiple dynamic responses evolved through microvascular propagation of ischemic and peri-ischemic regions and astrocyte nourishment in treatment groups [51]. In the NVU, on one hand, astrocytes protect neurons but are a danger to them. Our study provides evidence that Scu enhances neuronal survival and may improve special neurons glucose metabolism of cerebral ischemia [52, 53]. Moreover, Scu increased 18F-FDG uptake of neurons might be through facilitative expressing of GFAP-positive cells [54]. On the other hand, focal cerebral ischemia preferentially affects neurons distant from their neighboring microvessels [55]. In this study, the number of astrocytic GFAP-positive cells increased in all treatment groups and CIRU group compared to the sham group. Furthermore, 50 mg/kg of Scu not only increased glucose metabolism of neurons at the early stage of cerebral damage but also increased expression of NeuN induced by cerebral ischemia. Scu-50 may have potential to the prevention and treatment of stroke compared with Scu-25 and Scu which have shown anti-ischemia reperfusion activity in a dose-dependent manner. 

Nimodipine, a calcium channel blocker, originally developed for use in cardiovascular disorders. Our data indicated that nimodipine is recognized as an effective drug against cerebral ischemia but minor, when we set nimodipine previously as a positive control group, and therefore, we speculated that Scu might have potential protective effect by inhibiting influx of calcium ion against cerebral ischemia.

## 5. Conclusion 

This study demonstrated the metabolic recovery after high-dose Scu treatment by ^18^F-FDG microPET imaging and the neuroprotective effects of Scu in a rat model of cerebral ischemia. 

## Figures and Tables

**Table 1:** Values of five groups of neurological deficit after cerebral ischemia/reperfusion in different days (mean ± SEM).

Groups	*n*	Neurologic score				
		Day 0	Day 7	Day 14	Day 21	Day 28
Sham	6	0 ± 0	0 ± 0	0 ± 0	0 ± 0	0 ± 0
CIRU	6	12.00* ± 0.58	11.33* ± 0.33	10.33* ± 0.33	9.33* ± 0.33	7.67* ± 0.67
Scu-50	6	12.67* ± 0.88	7.67*** ± 0.33	7.67*** ± 0.33	6.33*** ± 0.33	2.67*** ± 0.33
Scu-25	6	12.33* ± 0.33	9.67** ± 0.33	8.67** ± 0.33	7.33*** ± 0.33	5.67** ± 0.33
Nimodipine	6	11.67* ± 0.33	10.33 ± 0.33	9.33 ± 0.33	7.67** ± 0.33	5.67** ± 0.33

Values are mean ± SD.

*P < 0.01; significantly higher compared with values of sham group.

**P < 0.05; significantly lower compared with values of CIRU group.

***P < 0.01; significantly lower compared with values of CIRU group.

**Table 2:** Infarct volume of cerebral ischemia after cerebral IR with TTC stain at the day 14 (mean ± SEM, mm^3^).

Group	*n *	Infarct volume
Sham	3	0.0 ± 0.00
CIRU	3	244.3* ± 7.76
Scu-50	3	56.0** ± 2.00
Scu-25	3	74.3^∗∗,w^ ± 3.51
Nimodipine	3	98.3** ± 3.06

Values are mean ± SD.

*P < 0.01; significantly higher compared with values of sham group.

**P < 0.01; significantly lower compared with values of CIRU group.

^w^ *P* < 0.01; significantly lower compared with values of Scu-25 group.

**Table 3:** Effects on glucose metabolism after cerebral IR in different days (mean ± SEM, ratio of lesion to cerebellum).

Groups	*n*	Glucose metabolism of rat brain using ^18^F-FDG microPET				
		Day 0	Day 7	Day 14	Day 21	Day 28
Sham	6	0.96 ± 0.02	0.91 ± 0.03	0.85 ± 0.03	0.99 ± 0.03	1.08 ± 0.03
CIRU	6	0.76* ± 0.03	0.67* ± 0.03	0.65* ± 0.03	0.60* ± 0.04	0.62* ± 0.04
Scu-50	6	0.75^∗w^ ± 0.02	0.71^∗w^ ± 0.04	0.74** ± 0.01	0.75** ± 0.04	0.76** ± 0.03
Scu-25	6	0.54* ± 0.03	0.49* ± 0.05	0.45* ± 0.02	0.46* ± 0.04	0.41* ± 0.04
Nimodipine	6	0.63* ± 0.03	0.44* ± 0.03	0.43* ± 0.05	0.40* ± 0.03	0.51* ± 0.02

Values are mean ± SD.

*P < 0.01; significantly higher compared with values of sham group.

**P < 0.01; significantly higher compared with values of CIRU group.

^w^ *P* > 0.05; compared with values of CIRU group.

**Table 4:** The expression of NVU immunoreactive (mean ± SEM, numbers of positive cells/field of vision).

Group	*n*	vWF	GFAP	NeuN
Sham group	3	7.33 ± 2.51	2222.24 ± 481.44	46.67 ± 8.74
CIRU group	3	11.33* ± 2.52	2936.06 ± 703.33	47.67 ± 8.14
Scu-50 group	3	13.0* ± 1.00	3438.27* ± 805.82	75.00^∗∗,w,∗∗∗^ ± 6.08
Scu-25 group	3	10.0* ± 1.00	3509.09* ± 281.83	48.33 ± 5.51
Nimodipine group	3	14.0* ± 1.73	3016.93 ± 113.45	59.67 ± 8.33

Values are means ± SD.

*P < 0.05; significantly lower compared with values of sham group.

**P < 0.01; significantly higher compared with values of Scu-25 group.

***P < 0.001; significantly higher compared with values of CIRU group.

^w^ *P* < 0.05; significantly higher compared with values of nimodipine group.
